# Nonnegligible Seroprevalence and Predictors of Murine Typhus, Japan

**DOI:** 10.3201/eid3002.230827

**Published:** 2024-02

**Authors:** Kentaro Iwata

**Affiliations:** Kobe University Hospital, Kobe, Japan

**Keywords:** murine typhus, *Rickettsia typhi*, seroprevalence, bacteria, Rickettsia, vector-borne infections, zoonoses, Japan

**To the Editor:** I was impressed by the recent publication by Aita et al. who reported a surprisingly high seroprevalence rate for *Rickettsia typhi* within resident populations on Honshu Island, Japan (*1*). The authors pointed out the possibility of murine typhus reemergence in Japan, where the disease has been reported only sporadically (*2*). However, the conclusions might be premature because the study was cross-sectional, and only 1 timepoint was evaluated. Many cases of murine typhus could have occurred in the distant past, which might not be reflected in this type of study. A previous study in Spain showed a high incidence rate in patients who were much younger (mean age of ≈46 years) (*3*) than those reported in this study (mean age of 67 years). A study in Greece showed frequent epidemiologic links to flea exposure (*4*), but the study in Japan did not investigate this apparent risk factor. I do not believe that age-related differences in flea exposures exist in Japan; hence, it is likely that exposures might have occurred in the past, when persons in Japan had a lower standard of hygiene. According to another study, the median half-life of *R. typhi* IgG was 177 days, and the median IgG titer was 800 at day 365 postinfection, suggesting long-lasting seropositivity (*5*). Therefore, the relatively stringent cutoff value of *R. typhi* serology in this study (*1*) could have overestimated the prevalence. To demonstrate that murine typhus is indeed a reemerging disease in Japan, further actual cases in Japan need to be identified, or similar seroprevalence studies should be repeated in other regions to determine trends in *R. typhi* seropositivity.
